# Antitumor and HIV-1 Reverse Transcriptase Inhibitory Activities of a Hemagglutinin and a Protease Inhibitor from Mini-Black Soybean

**DOI:** 10.1155/2011/851396

**Published:** 2011-03-06

**Authors:** Xiu Juan Ye, Tzi Bun Ng

**Affiliations:** School of Biomedical Sciences, Faculty of Medicine, The Chinese University of Hong Kong, Shatin New Territories, Hong Kong

## Abstract

Protease inhibitors (PIs) and hemagglutinins are defense proteins produced by many organisms. From Chinese mini-black soybeans, a 17.5-kDa PI was isolated using chromatography on Q-Sepharose, SP-Sepharose, and DEAE-cellulose. A 25-kDa hemagglutinin was purified similarly, but using Superdex 75 instead of DEAE-cellulose in the final step. The PI inhibited trypsin and chymotrypsin (IC_50_ = 7.2 and 8.8 *μ*M). Its trypsin inhibitory activity was stable from pH 2 to pH 13 and from 0°C to 70°C. The hemagglutinin activity of the hemagglutinin was stable from pH 2 to pH 13 and from 0°C to 75°C. The results indicated that both PI and hemagglutinin were relatively thermostable and pH-stable. The trypsin inhibitory activity was inhibited by dithiothreitol, signifying the importance of the disulfide bond to the activity. The hemagglutinating activity was inhibited most potently by D (+)-raffinose and N-acetyl-D-galactosamine, suggesting that the hemagglutinin was specific for these two sugars. Both PI and hemagglutinin inhibited HIV-1 reverse transcriptase (IC_50_ = 3.2 and 5.5 *μ*M), proliferation of breast cancer cells (IC_50_ = 9.7 and 3.5 *μ*M), and hepatoma cells (IC_50_ = 35 and 6.2 *μ*M), with relatively high potencies.

## 1. Introduction

Defense proteins including protease inhibitors are produced by a variety of organisms including animals [1] and plants [2–16]. Plants produce an array of defense proteins to combat noxious pathogens and predators. These defense proteins constitute a heterogeneous repertoire that comprises protease inhibitors [1–16], hemagglutinins [17], antifungal proteins [18], and ribosome inactivating proteins [19]. The defense proteins exert a diversity of actions such as immunomodulatory, antitumor/antiproliferative, and antiviral activities [2, 13–16]. Among the protease inhibitors, trypsin inhibitors represent a frequently studied group. Hemagglutinins are also a subject of intensive investigation. 

 The seeds of leguminous plants are abundant in proteins which may include some of the aforementioned defense proteins. The mini-black cultivar of soybean (*Glycine soja)* has not been examined previously. An extract of this bean exhibits protease inhibitory and hemagglutinating activities. Since different cultivars of the same species may produce different proteins, we undertook the present study to isolate and characterize a protease inhibitor and a hemagglutinin from the mini-black soybean. 

 Soybean has many beneficial effects and can ameliorate some diseases [20–22]. An aqueous extract of soybean *Glycine max* exerted a significant antihyperglycemic action in alloxan-induced diabetic mice [23]. Using monocyte/macrophage-like cell models [24], it has been demonstrated that a combination of avocado/soybean unsaponifiables and chondroitin sulfate can inhibit cytokine (TNF-*α* and IL-1*β*) expression and prostaglandin E_2_ production. Osteoarthritis is characterized by inflammation and elevated production of proinflammatory mediators such as cytokines and prostaglandin E2. Macrophage-like cells in synovial tissue produce these mediators which induce cartilage-degrading enzymes. Hence, it is possible to make use of soybeans to inhibit osteoarthritis.

Black soybean has long been used in Chinese traditional medicine, as a detoxifier, an anti-inflammatory drug and a blood nutrient and for promoting urination. It suppresses the growth of transplantable human bladder carcinoma and tumor angiogenesis in mice [25], exhibits antioxidant activity, inhibits low density lipoprotein oxidation [26], and can be used for treating an ophthalmic disorder associated with retinal pigment epithelium disturbance [27]. The polysaccharides prepared from black soybeans [28] can also be used to stimulate cellular immunity by augmenting the production of cytokines and in preventing or treating leukemia. It is beneficial to the health of patients who have been infected by pathogens such as HIV, by elevating the blood cell number and enhancing the immune system [29]. Black soybean is more effective than yellow soybean in preventing menopausal symptoms. Ovariectomized menopausal rats fed on black soybeans demonstrated a significantly greater reduction in blood-cholesterol concentration, compared to rats fed on yellow soybeans. Consumption of black soybeans protects against bone loss in ovariectomized rats by inhibiting bone turnover and bone resorption [30]. Black soybean peptides when applied in vivo and in vitro reduce endoplasmic reticulum stress and improve insulin resistance [31], whilst anthocyanins from black soybean seed coats stimulate wound healing in fibroblasts and keratinocytes and prevent inflammation in endothelial cells [32]. They preferentially inhibit tumor necrosis factor-alpha-mediated induction of vascular cell adhesion molecule-1 over intercellular adhesion molecule-1 through the regulation of transcription factor genes binding to DNA sequence GATA and interferon regulatory transcription factor-1 [33]. The anthocyanins inhibit UVB-induced inflammatory cylooxygenase-2 gene expression and PGE_2_ production through regulation of the nuclear factor-kappaB and phosphatidylinositol 3-kinase/Akt pathway [34]. They also exert hypolipidemic and antiobesity actions [35]. Red soybean anthocyanins demonstrate an antitumor action [36].

 Since black soybean has medicinal properties and its activities are reportedly more potent than those of yellow soybean, we undertook the present study to isolate a hemagglutinin and a protease inhibitor with antitumor and HIV-1 reverse transcriptase inhibitory activities from mini-black soybean which is a special type of black soybean.

## 2. Materials and Methods

### 2.1. Materials

 Chinese mini-black soybeans (*Glycine soja* cv. “Mini-black Soybean”) from China (100 g) were deposited in laboratory 302, School of Biomedical Sciences, The Chinese University of Hong Kong (CUHK) under voucher number LB302, after authentication by Professor Shiuying Hu, Honorary Professor of Chinese Medicine, CUHK. SP-Sepharose, Q-Sepharose, and Superdex 75 HR10/30 columns and Purifier were bought from GE Healthcare (Hong Kong). DEAE-cellulose, Trizma base (98% purity), NaCl (99% purity), and NH_4_OAc (98% purity) were obtained from Sigma Chemical Co., St. Louis, Missouri, USA.

### 2.2. Isolation of Protease Inhibitor and Hemagglutinin

 The beans were extracted with distilled water (10 mL/g) at room temperature in a Waring blender for 10 minutes, followed by centrifugation at 13000 g and 4°C for 30 minutes. Tris-HCl buffer (pH 7.4, 1 M) was added to the resulting supernatant until the final concentration of Tris attained 20 mM. The supernatant was then loaded on a 5 cm × 20 cm column of Q-Sepharose in 20 mM Tris-HCl buffer (pH 7.4). After removal of unabsorbed proteins, the column was eluted with 0.2 M NaCl added to the Tris-HCl buffer. The fraction eluted with 0.2 M NaCl was dialyzed extensively against distilled water, and 1 M NH_4_OAc buffer (pH 4.5) was added until the NH_4_OAc concentration reached 100 mM. This was followed by ion exchange chromatography on a 2.5 cm × 30 cm column of SP-Sepharose in the same buffer. After removal of unabsorbed proteins, the column was eluted with a linear 0–0.5 M NaCl gradient (total volume 650 mL) added to the NH_4_OAc buffer. The protease inhibitor-enriched fraction SP1 was eluted with the 0–0.25 M NaCl gradient, and the hemagglutinin-enriched fraction SP3 was eluted with the 0.175–0.5 M NaCl gradient. Both fractions were dialyzed extensively against distilled water at 4°C overnight. In the case of the protease inhibitor-enriched fraction, the Tris concentration of the fraction was adjusted to 20 mM. This was followed by ion exchange chromatography on a 2.5 cm × 30 cm column of DEAE-cellulose in 20 mM Tris-HCl buffer (pH 7.4). After removal of unabsorbed proteins, the column was eluted sequentially with a 0–0.5 M NaCl gradient (total volume 480 mL) added to the Tris-HCl buffer. Fraction D2, which was eluted with a 0.31–0.44 M NaCl gradient, was dialyzed against distilled water. D2 represented purified black soybean protease inhibitor. Hemagglutinin-enriched fraction SP3 was subjected to FPLC (fast protein liquid chromatography)-gel filtration on a Superdex 75 HR10/30 column using an AKTA Purifier.

### 2.3. Electrophoresis, Molecular Mass Determination, and N-terminal Amino Acid Sequence Analysis

 The purified protease inhibitor/hemagglutinin was subjected to sodium dodecyl sulfate-polyacrylamide gel electrophoresis (SDS-PAGE) for molecular mass determination. After electrophoresis, the gel was stained with Coomassie Brilliant Blue. The molecular mass of the isolated protein was determined by comparison of its electrophoretic mobility with those of molecular mass marker proteins from GE Healthcare. Gel filtration on an FPLC-Superdex 75 column, which had been calibrated with molecular mass markers (GE Healthcare), was conducted to determine the molecular mass of the protein. The N-terminal sequence of the protein was determined by using a Hewlett-Packard HP G1000A Edman degradation unit and a HP 1000 HPLC System.

### 2.4. Trypsin-Inhibitory and Chymotrypsin-Inhibitory Activities of Isolated Protease Inhibitor

Trypsin activity was determined by using casein from Sigma as substrate [35]. The assay for trypsin-inhibitory activity was carried out by addition of the test sample to 160 *μ*L of a 1% casein solution in 0.1 M Tris-HCl buffer (pH 7.4). Bovine pancreatic trypsin from Sigma (20 *μ*L of a 0.5 mg/mL solution) was then added and the mixture was incubated at 37°C for 15 minutes before 0.4 mL 5% (w/v) trichloroacetic acid was added to terminate the reaction. After centrifugation, the absorbance of the supernatant, which reflects the amount of casein fragments, was measured at 280 nm.

 The isolated trypsin inhibitor (2.4 *μ*M) was incubated with dithiothreitol (DTT) from Sigma, at final concentrations of 6 mM, 32 mM, and 64 mM for 25 min at 37°C. For comparison, soybean trypsin inhibitor (Sigma) (2.88 *μ*M) was similarly treated. The reaction was terminated by adding iodoacetamide at twice the amount of thiol functions contained in each DTT concentration. The remaining trypsin-inhibitory activity was measured at pH 8 as described above. The highest iodoacetamide concentration used in the test was devoid of any effect on the activity of trypsin and the trypsin-inhibitory activity of the isolated trypsin inhibitor and soybean trypsin inhibitor [8]. 

 Chymotrypsin activity was determined by using N-*α*-benzoyl-L-tyrosyl ethyl ester hydrochloride (BTEE) from Sigma as substrate and bovine pancreatic chymotrypsin (USB Corporation, Ohio, USA) [12]. When BTEE was used as substrate, 25 *μ*L of a serial concentration of purified trypsin inhibitor was incubated with 25 *μ*L chymotrypsin (1 mg/mL in 50 mM Tris-HCl containing 0.2 M CaCl_2_, pH 7.6) for 5 min at 37°C. The residual chymotrypsin activity was measured by adding 1.45 mL 0.25 mM N-*α*-benzoyl-L-arginine ethyl ester (BAEE) as substrate. After immediate mixing by inversion the increase in A253 was recorded for 5 min. Reactions without addition of test samples were used as positive control.

### 2.5. Hemagglutinating Activity of Isolated Hemagglutinin

Fifty microliters of a serial two-fold dilution of the hemagglutinin solution in a microtiter U-plate were mixed with 50 *μ*L of a 2% suspension of rabbit red blood cells in phosphate-buffered saline (pH 7.2) at 20°C. The results were read after about 1 h, when the blank had fully sedimented. One hemagglutination unit is defined as the reciprocal of the highest dilution exhibiting hemagglutination whilst specific activity is the number of hemagglutination units/mg protein [37].

 Serial twofold dilutions of sugar (melibiose, L-arabinose, D(+)-glucosamine, glucuronic acid, D(+)-xylose, D-galacturonic acid, *α*-lactose, D(+)-raffinose, mannitol, D(+)-galactose, D-glucose, D(+)-fucose, N-acetyl-D-galactosamine, D(+)-mannose, alpha-methyl-D-glucoside, D-galactonic acid, maltose, D-L-rhamnose, and xylitol from Sigma) samples were prepared in phosphate buffered saline (PBS). All of the dilutions were mixed with an equal volume (25 *μ*L) of a solution of the hemagglutinin with 3 hemagglutination units. The mixture was allowed to stand for 30 min at room temperature and then mixed with 50 *μ*L of a 2% rabbit erythrocyte suspension. The minimum concentration of the sugar in the final reaction mixture, which completely inhibited 3 hemagglutination units of the hemagglutinin preparation, was calculated [37].

### 2.6. Thermostability of Hemagglutinin and Protease Inhibitor

The hemagglutinin samples and trypsin inhibitor samples were kept separately at different temperatures (10°C to 100°C, at 10°C intervals) for 30 min. The hemagglutinating activity and trypsin-inhibitory activity were determined after returning to room temperature [37–40].

### 2.7. pH Stability of Hemagglutinin

The hemagglutinin samples were kept separately at different pH values (2 to 13) for 30 min. After the pH had been adjusted to pH 7, the hemagglutinating activity was determined [37–40].

### 2.8. Antiproliferative Activity of Isolated Protease Inhibitor and Hemagglutinin on Tumor Cell Lines

 Breast cancer (MCF-7) cells and hepatoma (HepG2) cells from American Type Tissue Collection were suspended in RPMI medium containing 10% fetal bovine serum, 100 U/mL penicillin, and 100 *μ*g/mL streptomycin (Gibco) and adjusted to a cell density of 2 × 10^4^ cells/mL. A 100 *μ*L aliquot of this cell suspension was seeded to a well of a 96-well plate, followed by incubation for 24 hours. Different concentrations of the protease inhibitor or hemagglutinin in 100 *μ*L complete RPMI medium were then added to the wells and incubated for 72 hours at 37°C. After 72 hours, 20 *μ*L of a 5 mg/mL solution of (3-[4,5-dimethylthiazol-2-yl]-2,5-diphenyltetrazolium bromide) (MTT) in phosphate buffered saline was spiked into each well and the plates were incubated for 4 hours. The plates were then centrifuged at 324× g for 5 minutes. The supernatant was carefully removed, and 150 *μ*L of dimethyl sulfoxide was added in each well to dissolve the MTT-formazan at the bottom of the wells. After 10 minutes, the absorbance at 590 nm was measured by using a microplate reader [18]. The defensin-like antifungal peptide sesquin from the bean *Vigna sesquipedalis* [18] was used as a positive control. Green lentil trypsin inhibitor [9] was used as a negative control in the assay.

### 2.9. Ability of Isolated Protease Inhibitor and Hemagglutinin to Inhibit HIV-1 Reverse Transcriptase Inhibitory Activity

The assay for ability to inhibit HIV reverse transcriptase inhibitory activity was carried out according to instructions supplied with the assay kit from Boehringer Mannheim (Germany). The assay takes advantage of the ability of reverse transcriptase to synthesize DNA, starting from the template/primer hybrid poly (A) oligo (dT) 15. The digoxigenin- and biotin-labeled nucleotides in an optimized ratio are incorporated into the DNA molecule, which is freshly synthesized by the reverse transcriptase (RT). The detection and quantification of synthesized DNA as a parameter for RT activity follow a sandwich ELISA protocol. Biotin-labeled DNA binds to the surface of microtiter plate modules that have been precoated with streptavidin. In the next step, an antibody to digoxigenin, conjugated to peroxidase, binds to the digoxigenin-labeled DNA. In the final step, the peroxidase substrate is added. The peroxidase enzyme catalyzes the cleavage of the substrate, producing a colored reaction product. The absorbance of the sample at 405 nm can be determined using a microtiter plate (ELISA) reader and is directly correlated to the level of RT activity. A fixed amount (4–6 ng) of recombinant HIV-1 reverse transcriptase was used. The inhibitory activity of the protease inhibitor/hemagglutinin was calculated as percent inhibition compared with the control without the protein [18]. The leguminous defensin-like protein sesquin was used as a positive control [18] and the antifungal protein mungin [41] as a negative control.

### 2.10. Antifungal Activity of Isolated Hemagglutinin and Protease Inhibitor

The assay of the isolated protease inhibitor/hemagglutinin for antifungal activity toward *Botrytis cinerea, Mycosphaerella arachidicola*, and *Fusarium oxysporum*, which are plant pathogens, was carried out using 90 × 15 mm petri plates containing 10 mL of potato dextrose agar. After the mycelial colony had developed, sterile blank paper disks (0.625 cm in diameter) were placed at a distance of 0.5 cm away from the rim of the mycelial colony. An aliquot of a solution of the protease inhibitor/hemagglutinin was added to a disk. After the plates had been incubated at 25°C for 72 hours, mycelial growth had enveloped disks containing the control and had formed crescents of inhibition around disks containing samples with antifungal activity. The leguminous defensin-like peptide sesquin was employed as a positive control [18] and emperor banana lectin [37] as a negative control.

## 3. Results

### 3.1. Isolation of Protease-Inhibitor and Hemagglutinin

**Figure 1:**
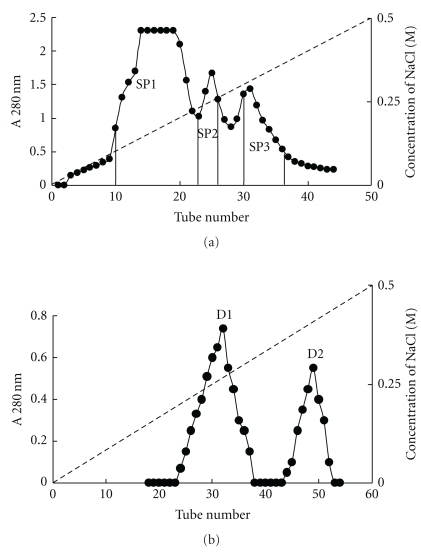
(a) Ion exchange chromatography on a SP-Sepharose column (2.5 cm × 30 cm). Sample: fraction of the mini-black soybean extract adsorbed on Q-Sepharose and subsequently eluted by 0.2 M NaCl in 20 mM Tris-HCl buffer. The dotted slanting line represents the linear NaCl concentration gradient (0–0.5 M) in 100 mM NH_4_OAc buffer (pH 4.5) used to elute the absorbed proteins from the column. Unabsorbed proteins eluted with 100 mM NH_4_OAc buffer (pH 4.5) are not shown. Hemagglutinating activity was observed in fraction SP3. Trypsin-inhibitory activity was found in fraction SP1. Fraction size: 13 mL, flow rate: 30 mL/h. Conc. Of NaCl (M- - -), and absorbance at 280 nm (●). The vertical lines in the elution profile denote the pooling of eluate into SP1, SP2, and SP3. (b) Ion exchange chromatography on a DEAE-cellulose column (2.5 cm × 30 cm). Sample: Fraction SP1, the first adsorbed fraction from SP-Sepharose. Adsorbed proteins were eluted from the column by using a linear NaCl concentration gradient (0–0.5 M) in 20 mM Tris-HCl buffer (pH 7.4) represented by the dotted slanting line across the chromatogram. Trypsin-inhibitory activity was observed only in fraction D2. Unabsorbed proteins eluted with 20 mM Tris-HCl buffer (pH 7.4) are not shown. Fraction size: 6 mL, flow rate: 30 mL/h, Conc of NaCl (M- - -), and absorbance at 280 nm (●).

**Figure 2:**
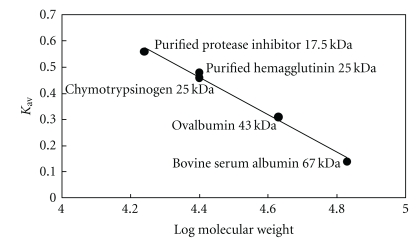
Standard curve for the molecular weight determination of the purified protease inhibitor and hemagglutinin from mini-black soybean using Superdex 75HR 10/30 chromatography on Superdex 75HR 10/30.

**Figure 3:**
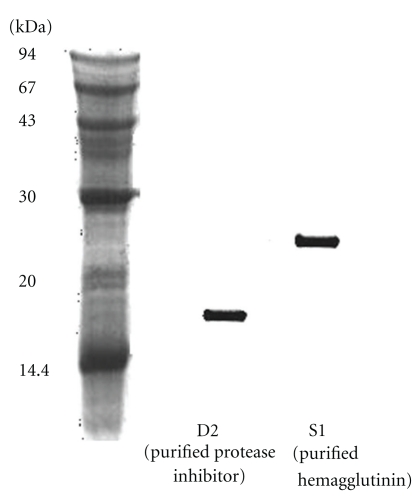
Results of SDS-PAGE after staining with Coomassie Brilliant Blue. Left lane: markers from GE Healthcare including phosphorylase b (94-kDa), bovine serum albumin (67-kDa), ovalbumin (43-kDa), carbonic anhydrase (30-kDa), soybean trypsin inhibitor (20-kDa), and *α*-lactalbumin (14.4-kDa). Middle lane: fraction D2 with trypsin-inhibitory activity from DEAE-cellulose column chromatography representing purified protease inhibitor. Right lane: fraction S1 from Superdex 75 column representing purified mini-black soybean hemagglutinin.

**Figure 4:**
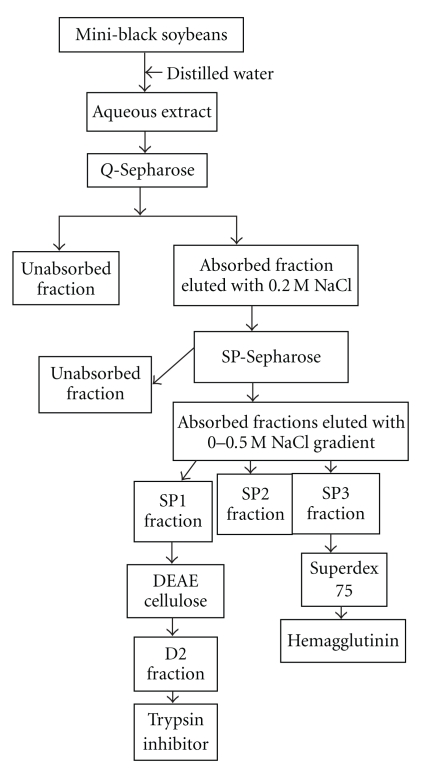
Flow chart summarizing the procedure used for isolating protease inhibitor and hemagglutinin from mini-black soybean.

When the extract of mini-black soybeans was chromatographed on Q-Sepharose, trypsin-inhibitory activity, and hemagglutinating activity resided in the adsorbed fraction eluted by the linear concentration gradient of 0 to 0.2 M NaCl, but not in the unabsorbed fraction. This adsorbed fraction was subsequently resolved on SP-Sepharose into an unabsorbed fraction (devoid of trypsin-inhibitory and hemagglutinating activities) and several adsorbed fractions, SP1, SP2, and SP3. Trypsin-inhibitory activity was detected in the largest adsorbed fraction SP1, and hemagglutinating activity was found in fraction SP3 which was much smaller in size (Figure 1(a)). Fraction SP1 was subsequently fractionated on DEAE-cellulose into an inactive unabsorbed fraction and two adsorbed fractions, D1 and D2 (Figure 1(b)). Trypsin-inhibitory activity was located only in fraction D2, which represented purified trypsin inhibitor. The molecular mass of D2 as determined by gel filtration on Superdex 75 was 17.5-kDa (Figure 2). There was a 47-fold purification of trypsin-inhibitory activity (Table 1). Hemagglutinating activity was adsorbed on Q-Sepharose and eluted with 0.2 M NaCl in 20 mM Tris-HCl buffer (pH 7.4). The fraction was subsequently adsorbed on SP-Sepharose and eluted in fraction SP3. Fraction SP3 appeared as a single peak upon gel filtration on Superdex 75. The peak had a molecular mass of 25-kDa (Figure 2) and appeared as a single 25-kDa band in SDS-PAGE (Figure 3). The purified protease inhibitor demonstrated a molecular mass of 17.5-kDa in SDS-PAGE (Figure 3). A summary of purification of the hemagglutinin is provided in Table 2.  Figure 4 is a flow chart illustrating the scheme for concurrent isolation of the protease inhibitor and hemagglutinin.

### 3.2. N-Terminal Sequences of Trypsin Inhibitor and Lectin

The data are shown in Table 3. Considerable homology to their counterparts from soybean is observed.

### 3.3. Biological Activities of Protease Inhibitor and Hemagglutinin

**Figure 5:**
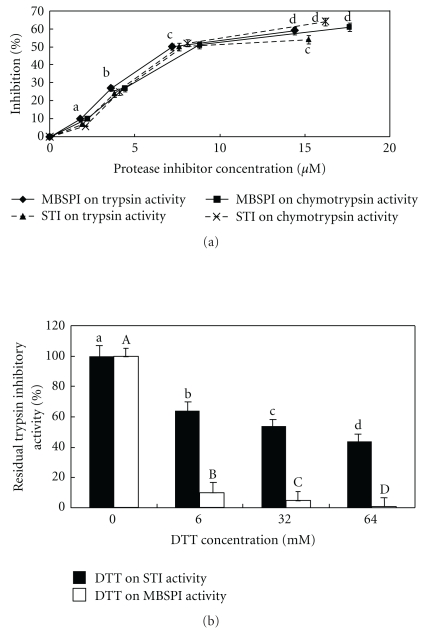
(a) Determination of IC_50_ values of trypsin- and chymotrypsin-inhibitory activities of mini-black soybean protease inhibitor (MBSTI) and soybean trypsin inhibitor (STI). Results are means ± SD (*n* = 3). The protease inhibitor was incubated with bovine pancreatic trypsin and casein (for assay of trypsin inhibitory activity) or with N-*α*-benzoyl-L-tyrosyl ethyl ester hydrochloride (for assay of chymotrypsin inhibitory activity) and bovine pancreatic chymotrypsin for 15 minutes in 0.1 M Tris-HCl buffer (pH 7.4) before addition of 5% trichloroacetic acid. The reaction mixture was centrifuged and OD280 of the supernatant containing the tryptic fragments of casein was read. Background values were determined and subtracted before % inhibition values were calculated. Data points bearing the same letter represent statistically significant difference (*P* < .05) when the data were analyzed by ANOVA followed by Duncan's multiple range test. (b) Effect of dithiothreitol (DTT) on trypsin-inhibitory activity of 2.4 *μ*M mini-black soybean (MBSPI) and 2.8 *μ*M trypsin inhibitor from soybean (STI) after incubation at 37°C for 25 minutes. Results are means ± SD (*n* = 3). The IC_50_ of DTT was about 30 mM on MBSPI and lower than 10 mM on STI. The protease inhibitor (1.8 mM) was incubated with dithiothreitol at various concentrations before termination of reaction with iodoacetamide and determination of remaining trypsin-inhibitory activity. Background values were determined and subtracted before % inhibition values were calculated. Data points bearing the same letter represent statistically significant difference (*P* < .05) when the data were analyzed by ANOVA followed by Duncan's multiple range test.

The isolated protease inhibitor inhibited trypsin with an IC_50_ of 7.2 ± 0.5 *μ*M (mean ± SD, *n* = 3) (Figure 5(a)), and chymotrypsin with an IC_50_ of 8.8 ± 0.2 *μ*M (mean ± SD, *n* = 3) (Figure 5(a)). Dithiothreitol inhibited the trypsin-inhibitory activity of the protease inhibitor in a dose-dependent manner (Figure 5(b)). The trypsin-inhibitory activity of the protease inhibitor and the hemagglutinating activity of the lectin were stable from pH 2 to pH 13 (data not shown). Activity was fully preserved from 0°C to 70°C for the protease inhibitor and from 0°C to 75°C for the hemagglutinin (data not shown). 

**Figure 6:**
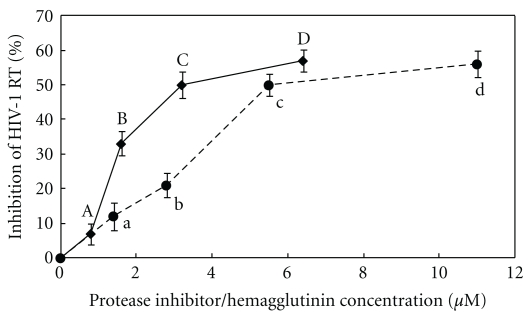
Inhibitory effect of mini-black soybean protease inhibitor (♦) and hemagglutinin (●) on HIV-1 reverse transcriptase activity. Results are means ± SD (*n* = 3). The inhibitory activities were determined with an ELISA kit from Boehringer Mannheim. Background values were determined and subtracted before % inhibition values were calculated. Data points bearing the same letter represent statistically significant difference (*P* < .05) when the data were analyzed by ANOVA followed by Duncan's multiple range test.

**Figure 7:**
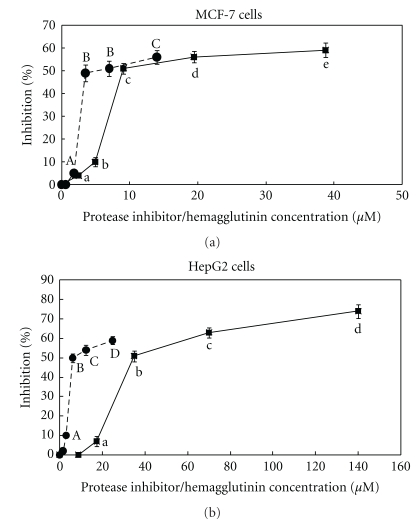
(a) Antiproliferative effect of mini-black soybean protease inhibitor (♦) and hemagglutinin (●) on MCF-7 cells. Results are presented as means ± SD (*n* = 3). The inhibitor or lectin was incubated with 2 × 10^−3^ MCF-7 cells in a 96-well plate for 72 hours prior to incubation with MTT for 4 hours. The plate was centrifuged and the supernatant removed before addition of DMSO to dissolve the MTT-formazan at the bottom of the well. OD_590 nm_ was then measured after 10 minutes. Background values were determined and subtracted before % inhibition values were calculated. Data points bearing the same letter represent statistically significant difference (*P* < .05) when the data were analyzed by ANOVA followed by Duncan's multiple range test. (b) Antiproliferative effect of mini-black soybean protease inhibitor (♦) and hemagglutinin (●) on HepG2 cells. Results are means ± SD (*n* = 3). The inhibitor or the hemagglutinin was incubated with 2 × 10^−3^ HepG2 cells in a 96-well plate for 72 hours prior to incubation with MTT for 4 hours. The plate was centrifuged and the supernatant removed before addition of DMSO to dissolve the MTT-formazan at the bottom of the well. OD_590 nm_ was then measured after 10 minutes. Background values were determined and subtracted before % inhibition values were calculated. Data points bearing the same letter represent statistically significant difference (*P* < .05) when the data were analyzed by ANOVA followed by Duncan's multiple range test.

The protease inhibitor and the hemagglutinin inhibited HIV-1 reverse transcriptase with an IC_50_ of 3.2 ± 0.2 *μ*M and 5.5 ± 0.5 *μ*M (Figure 6), and suppressed proliferation of MCF-7 breast cancer cells with an IC_50_ of 9.7 ± 0.3 *μ*M and 3.5 ± 0.2 *μ*M (Figure 7(a)), and HepG2 hepatoma cells with an IC_50_ of 35 ± 0.3 *μ*M and 6.2 ± 0.3 *μ*M (Figure 7(b)), respectively. The hemagglutinating activity of the hemagglutinin was inhibited by sugars with the following ranking of potencies: D(+) – raffinose = N-acetyl-D-galactosamine ⋙ melibiose = L-arabinose > *α*-lactose > D(+)-glucosamine > glucuronic acid > D(+)-fucose (Table 4). 

**Figure 8:**
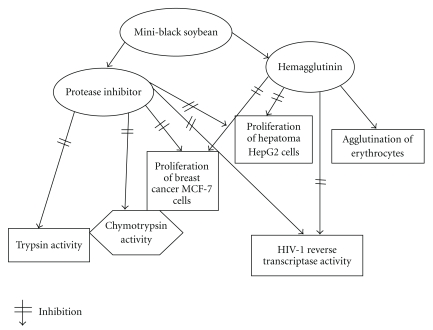
Biological activities of protease inhibitor and hemagglutinin from mini-black soybean.

Neither the protease inhibitor nor the hemagglutinin demonstrated antifungal activity when tested up to 100 *μ*M (data not shown). Biological activities which the isolated protease inhibitor and hemagglutinin have in common are shown in Figure 8.

### 3.4. Comparison of Hemagglutinin and Protease Inhibitor from Mini-Black Soybean with Those from Soybean

The comparison is shown in Tables 5 and 6. Some differences are noted between proteins from the two cultivars of soybean.

## 4. Discussion

We report herein the concurrent purification of a protease inhibitor and a hemagglutinin from the mini-black soybean. Both proteins are adsorbed on Q-Sepharose and SP-Sepharose but can be resolved from one another by using a linear NaCl concentration (0–0.5 M) gradient to elute adsorbed proteins from the SP-Sepharose column. The hemagglutinin is more strongly adsorbed on SP-Sepharose than the protease inhibitor. It requires only two steps, ion exchange chromatography on Q-Sepharose and SP-Sepharose, to purify the hemagglutinin since it appears as a single band in SDS-PAGE and a single peak in gel filtration after SP-Sepharose chromatography. In a previous investigation, simultaneous purification of a protease inhibitor and a hemagglutinin from *Pseudostellaria heterophylla* roots has been achieved. Both the protease inhibitor and the lectin are unabsorbed on DEAE-cellulose and adsorbed on CM-cellulose. They can be separated from one another by gel filtration on Superdex 75 in which the 36-kDa lectin appears as the first peak and the 20.5-kDa protease inhibitor is eluted as the second peak [38]. Similar to protease inhibitors from other leguminous seeds such as broad bean [2], mini-black soybean protease inhibitor is also a chymotrypsin inhibitor. The ability of the reducing agent dithiothreitol to reduce the trypsin-inhibitory activity of mini-black soybean protease inhibitor in a dose-dependent manner, as in the case of trypsin-inhibitor from papaya [8], revealed the importance of disulfide bonds to the trypsin-inhibitory activity. The ability of mini-black soybean protease inhibitor to inhibit proliferation of hepatoma and breast cancer cells is in compliance with earlier reports of the antitumor/antiproliferative activity of leguminous protease inhibitors [14–17]. The HIV-1 reverse transcriptase inhibitory activity of the protease inhibitor is also in agreement with previous findings on trypsin inhibitors from the broad bean [2] and wampee [11]. However, it is noteworthy that some trypsin inhibitors like those of lentil [9], *Vigna mungo* [39], and lily bulbs [40] are devoid of antiproliferative activity toward tumor cells and exhibit very weak or no HIV-1 reverse transcriptase inhibitory activity. The mechanism of the inhibitory action on the retroviral enzyme may be protein-protein interaction [42–44]. HIV-1 protease inhibits the homologous reverse transcriptase with this mechanism [44]. Unlike broad bean trypsin inhibitor [2] but similar to those of lentil [9], *Vigna mungo* [39], and lily bulbs [40], the protease inhibitor isolated in the present study does not suppress mycelial growth. It is a Kunitz-type trypsin inhibitor as evidenced by its molecular mass and N-terminal sequence.

 The hemagglutinin isolated in the present study is capable of potently inhibiting HIV-1 reverse transcriptase. Some hemagglutinins have been shown to suppress HIV replication [45] and reduce HIV infectivity [46] and infections [47] while other hemagglutinins inhibit the retroviral reverse transcriptase [48, 49]. The hemagglutinin also exerts its potent antiproliferative activity against hepatoma HepG2 and breast cancer MCF-7 cells, the findings are in line with previous reports on the antiproliferative/antitumor activity of other hemagglutinins [17, 47–56]. Lectins and lectin-containing extracts induce apoptosis [57] and regulate the expression of apoptotic genes in cancer cells [58, 59]. It is highly likely that mini-black soybean hemagglutinin exerts its antitumor effects on breast cancer MCF-7 cells with a mechanism similar to that employed by French bean hemagglutinin [58]. The hemagglutinin demonstrates a higher antiproliferative activity on hepatoma and breast cancer cells than the protease inhibitor. However, it manifests a weaker HIV-1 reverse transcriptase inhibitory activity than the protease inhibitor.

Results of the present investigation on a Chinese cultivar of black soybean show some differences from earlier reports on soybean trypsin inhibitor and soybean lectin. The early findings were on a Bowman-Birk type trypsin inhibitor with a molecular mass of about 8-kDa and a Kunitz-type trypsin inhibitor with a molecular mass of about 21-kDa [16]. However, only a single 17.5-kDa Kunitz-type inhibitor was found in the present study. Soybean lectin reported in the literature is specific for galactose. The hemagglutinin isolated in the present study has a sugar specificity toward both D(+)-raffinose and N-acetylgalactosamine. Both protease inhibitor and hemagglutinin isolated in the present study have potent HIV-1 reverse transcriptase inhibitory and antiproliferative activities with IC_50_ values mostly below 10 *μ*M. It appears that mini-black soybean may have health promoting effects.

 The two proteins isolated in the present study are devoid of antifungal activity. Some [2, 11] but not other [9, 12] protease inhibitors display antifungal activity. Similarly, to date only a small number of hemagglutinins with antifungal activity have been reported [60, 61].

 Different cultivars of the same plant species may produce different proteins. For instance, different cultivars of the pea *Pisum sativum* produce the ribosome inactivating protein pisavin [19] and the antifungal protein sativin [62], respectively. Different cultivars of the bean *Phaseolus vulgaris* including the pinto bean, flageolet bean, haricot bean, French bean, and red kidney bean produce different lectins [63]. From the Japanese black soybean [64], a Bowman-Birk trypsin inhibitor with antiproliferative activity but no Kunitz-type trypsin inhibitor has been isolated. Both types of trypsin inhibitor are produced by the soybean [2] but only a Kunitz-type trypsin inhibitor is elaborated by the mini-black soybean cultivar. The number and type of protease inhibitor produced by soybean depend on the cultivar. Hemagglutinins produced by different soybean cultivars may also be different. The results of the present investigation are in line with the aforementioned observation. 

 Antiviral activity against two respiratory illness viruses, human adenovirus type 1 and coxsackie virus B1, has been demonstrated in a hot-water extract of black soybean. In contrast, the hot-water extract of common yellow soybean exhibited much lower activity. Neither saponins nor anthocyanins account for the antiviral activity. A partially purified hydrophilic and anionic antiviral compound was obtained by gel filtration, reversed phase HPLC, and ion-exchange HPLC. It showed maximum absorption at 260 nm, indicating the presence of phenyl groups. Amino acid analysis and neutral sugar analysis disclosed that the antiviral compound is not a protein or glycoconjugate with neutral sugars [65]. Anthocyanidins are possible anti-inflammatory agents; however, further studies are required to determine required intake levels *in vivo* to exert antitumor effect [66]. The anticancer therapeutic potential of soy isoflavone genistein was reviewed by Ravindranath et al. [67]. 

 The aforementioned investigations conducted by other groups revealed the antiviral and antitumor activities of non-proteinaceous constituents of black soybean. The present study demonstrated that protein components of black soybean exhibit these activities. 

 Soybean trypsin inhibitor remains stable for 60 minutes in simulated gastric fluid. The bulk of soybean lectin is digested within 8 minutes. The results on simulated gastric fluid differ from those on simulated intestinal fluid. Soybean trypsin inhibitor, soybean lectin, and peanut lectin are stable for 60 minutes in simulated intestinal fluid [68]. The legume lectin *Griffonia simplicifolia* lectin II [69] and banana lectin [70] are trypsin-stable. Thus, it is likely that soybean trypsin inhibitor and soybean lectin are relatively stable after passage through the gastrointestinal tract.

In this investigation, a protease inhibitor and a hemagglutinin, with some differences in characteristics from soybean trypsin inhibitor and soybean lectin reported earlier, have been isolated using some protocol from a Chinese cultivar of soybean, the mini-black soybean. In traditional Chinese medicine, black soybean is more nutritious and reinvigorating than soybean. The finding of a hemagglutinin and a protease inhibitor from black soybean, both with relatively stable medicinal activities including HIV-1 reverse transcriptase inhibitory and antiproliferative activities, at least partially account for its use in traditional Chinese medicine.

## Figures and Tables

**Table 1:** Yields (from 50 g beans) and trypsin-inhibitory activities of various chromatographic fractions obtained during different steps of purification of mini-black soybean protease inhibitor.

Fraction	Total protein (mg)	Total trypsin-inhibitory activity (u)	Specific trypsin-inhibitory activity (u/mg)	Recovery of trypsin-inhibitory activity (%)	Fold of purification
Extract	9800	17640	1.8	100	1
Q	625	9375	15	53.1	8.3
SP1	242	8228	34	46.6	18.9
D2	53	4505	85	25.5	47.2

**Table 2:** Yields (from 50 g beans) and hemagglutinating activities of various chromatographic fractions obtained during different steps of purification of mini-black soybean hemagglutinin. HA: hemagglutinating.

Fraction	Total protein (mg)	Total HA activity (u)	Specific HA activity (u/mg)	Recovery of HA activity(%)	Fold of purification
Extract	9800	87220	8.9	100	1
Q	625	30010	48	34.4	5.3
SP3	23	5888	256	6.8	28.8
S	18	5526	307	6.3	34.5

**Table 3:** N-terminal sequences of protease inhibitor and hemagglutinin from mini-black soybean.

	Residue number	Sequence	Residue number
Kunitz-type trypsin inhibitor from mini-black soybean (*Glycine soja*)	1	GFVLDNQGNPLQNGGTYYLLSDITAFGGIRAAPTGNERYPLTVVQS	47

Trypsin inhibitor subtype B from yellow soybean (*Glycine max*)	1	DVLDNEGNPLQNGGTYYILSDITAFGGIRAAPTGNERYPLTVVQS	47

mini-black soybean hemagglutinin	1	AQTVSFSSWNKFVPKQPNILQGDAEVTSTGKLQLKAVKN	39

yellow soybean lectin	1	AETTSFSITK FVPDQKNLIFQGDAEVTSTGKLKLKAVKN	39

**Table 4:** Effect of various carbohydrates on hemagglutinating activity of mini-black soybean hemagglutinin. Initial hemagglutinating activity was 3 hemagglutinating units, +: hemagglutination in presence of sugar, −: no hemagglutination in presence of sugar.

Sugar (mM)	1.6	3.12	6.25	12.5	25	50	100	200
Melibiose	+	+	+	+	−	−	−	−
L-Arabinose	+	+	+	+	−	−	−	−
D(+)-Glucosamine	+	+	+	+	+	+	−	−
Glucuronic acid	+	+	+	+	+	+	−	−
D(+)-Xylose	+	+	+	+	+	+	+	+
D-Galacturonic acid	+	+	+	+	+	+	+	+
*α*-Lactose	+	+	+	+	+	−	−	−
D(+)-Raffinose	−	−	−	−	−	−	−	−
Mannitol	+	+	+	+	+	+	+	+
D(+)-Galactose	+	+	+	+	+	+	+	+
D-Glucose	+	+	+	+	+	+	+	+
D(+)-Fucose	+	+	+	+	+	+	−	−
N-Acetyl-D-Galactosamine	−	−	−	−	−	−	−	−
D(+)-Mannose	+	+	+	+	+	+	+	+
Alpha-Methyl-D-Glucoside	+	+	+	+	+	+	+	+
D-Galactonic acid	+	+	+	+	+	+	+	+
Maltose	+	+	+	+	+	+	+	+
D-L-Rhamnose	+	+	+	+	+	+	+	+
Xylitol	+	+	+	+	+	+	+	+

**Table 5:** Comparison of lectins from different cultivars of soybean.

	Soybean lectin	Chinese mini-black soybean hemagglutinin
Molecular weight	122 kDa	25 kDa
No. of subunits	4	1
Sugar specificity	N-acetylgalactosamine	D(+)-raffinose
pH stability	pH 2–13	pH 2–13
Thermostability	Up to 50°C	Up to 75°C
Antitumor activity	Present	HepG2 IC_50_: 6.2 *μ*M
		MCF-7 IC_50_: 3.5 *μ*M
Inhibitory activity of HIV-1 reverse transcriptase	Present	IC_50_: 5.5 *μ*M

**Table 6:** Comparison of trypsin inhibitors from different cultivars of soybean.

	Soybean trypsin inhibitor	Chinese mini-black soybean Protease inhibitor
Molecular weight	20 kDa	17.5 kDa
Monomeric/with subunits	Monomeric	Monomeric
pH stability	pH 3–13	pH 2–13
Thermostability	Not done	Up to 70°C
Trypsin inhibitory activity	IC_50_: 7.2 *μ*M	IC_50_: 8.8 *μ*M
Chymotrypsin inhibitory activity	IC_50_: 7.6 *μ*M	IC_50_: 7.9 *μ*M
Inhibition of trypsin inhibitor by DTT (IC_50_)	Lower than 10 mM	30 mM
